# Divergent skeletal muscle metabolite exchange in insulin‐like growth factor‐1‐stimulated myotubes and resistance‐exercised human muscle

**DOI:** 10.1113/EP094096

**Published:** 2026-07-02

**Authors:** Tim Havers, Moritz Eggelbusch, Marius Meinhold, Kira Sing, Rainer Okrojek, Anna Artati, Michael Witting, Dominik Lutter, Claire V. Kieker, Collin Starke, Tushar More, Karsten Hiller, Flemming Dela, Martin Schönfelder, André Kafka, Stephan Geisler, Henning Wackerhage

**Affiliations:** ^1^ TUM‐School of Medicine and Health Technical University of Munich Munich Germany; ^2^ Department of Fitness & Health IST University of Applied Sciences Düsseldorf Germany; ^3^ Department of Comparative Biosciences University of Wisconsin‐Madison Madison Wisconsin USA; ^4^ School of Veterinary Medicine University of Wisconsin‐Madison Madison Wisconsin USA; ^5^ Medical Clinic and Polyclinic I Rechts der Isar Hospital of the Technical University of Munich Munich Germany; ^6^ Metabolomics and Proteomics Core Helmholtz Zentrum München Neuherberg Germany; ^7^ Chair of Analytical Food Chemistry, TUM‐School of Life Sciences Technical University of Munich Freising‐Weihenstephan Germany; ^8^ Institute for Diabetes and Obesity, Helmholtz Diabetes Center Helmholtz Zentrum München, German Research Center for Environmental Health Neuherberg Germany; ^9^ German Center for Diabetes Research Neuherberg Germany; ^10^ Department of Biochemistry and Bioinformatics Technische Universität Braunschweig Braunschweig Germany; ^11^ Department of Biomedical Sciences, Faculty of Health and Medical Sciences University of Copenhagen Copenhagen Denmark

**Keywords:** anabolic reprogramming, arteriovenous metabolomics, C2C12 myotubes, skeletal muscle hypertrophy, Warburg‐like metabolism

## Abstract

Skeletal muscle hypertrophy requires a substantial nutrient influx for biomass accretion, but global metabolite exchange during growth is poorly characterized. Therefore, we profiled the metabolite uptake and release in insulin‐like growth factor‐1 (IGF‐1)‐stimulated C2C12 myotubes and human muscle 24 h after resistance exercise. Differentiated C2C12 myotubes were stimulated to grow with IGF‐1 (100 ng/mL, 24 h) or received vehicle control. To measure metabolite exchange, we analysed fresh and spent media by gas chromatography–mass spectroscopy metabolomics. In a second experiment, seven untrained adults (three males and four females; age 25.6 ± 3.2 years; body mass index 23.8 ± 2.8 kg/m^2^) performed single‐leg hypertrophy‐oriented resistance exercise, with the contralateral leg serving as the control. After 24 h, we obtained arteriovenous blood samples in the postabsorptive state and analysed plasma by untargeted liquid chromatography–mass spectroscopy metabolomics, characterizing the directionality of metabolite exchange across the human leg. In vitro, IGF‐1 increased uptake of serine, arginine and pyridoxamine, while enhancing lactate release (all *P* < 0.05), reflecting anabolic, Warburg‐like reprogramming. In vivo, 24 h postexercise there were modest global shifts (principal components analysis: PC1 8.8%, PC2 6.3% variance) and no significant essential amino acid uptake. Nominal differences (*P* < 0.05) included increased uptake of peptide‐related metabolites (acisoga and 2‐amino‐4‐CP) and α‐ketoglutarate, alongside release of C12:0 and C16:0 acylcarnitines. No in vivo differences persisted after false discovery rate correction. Although IGF‐1 stimulation in vitro promotes coordinated nitrogen‐rich metabolite uptake and lactate release, human muscle 24 h postexercise in a postabsorptive state is characterized by increased peptide turnover and lipid release rather than net amino acid uptake. This indicates limited substrate accumulation and net biomass accretion in the absence of exogenous nutrients.

## Abstract (highlights)

- **What is the central question of this study?**
  How does skeletal muscle growth, induced by insulin‐like growth factor‐1 (IGF‐1) stimulation in vitro and resistance exercise in humans, alter global metabolite exchange between muscle and the circulation? Does this process involve increased systemic nutrient uptake, especially of amino acids?
- **What is the main finding and its importance?**
  IGF‐1 induces anabolic, Warburg‐like metabolite uptake in myotubes, but human skeletal muscle 24 h after resistance exercise shows no net amino acid uptake in the postabsorptive state. This indicates that early postexercise muscle growth is not driven by increased systemic amino acid acquisition, highlighting context‐dependent metabolic reprogramming.

## 1 INTRODUCTION

Exercise is a powerful intervention for preserving health, preventing chronic disease and reducing mortality. Although early public health recommendations emphasized endurance exercise (American College of Sports Medicine, 1978; World Health Organization, 2004), current guidelines advocate a combination of endurance and resistance training (Bull et al., 2020; Piercy & Troiano, 2018). Resistance training confers many of the systemic health benefits of endurance exercise while providing additional advantages, including preservation of muscle mass and function with ageing, in addition to increased bone quality (Izquierdo et al., 2025; Pedersen & Saltin, 2015; Westcott, 2012).

Beyond musculoskeletal adaptations, resistance training also exerts clinically meaningful anti‐obesity and anti‐diabetes effects (Havers et al., 2025). In humans, modest increases in global muscle mass (∼2–3%) are associated with a ∼3% reduction in fat mass, improvements in glycaemic control, including lower glycosylated haemoglobin (∼4% relative reduction) and a ∼6% decline in fasting glucose levels (Havers et al., 2025). Experimental models support a causal link between muscle hypertrophy and metabolic health. Genetic or pharmacological stimulation of muscle hypertrophy induced, for instance, by myostatin deletion (Guo et al., 2009; McPherron & Lee, 2002) or constitutive Akt1 activation (Izumiya et al., 2008), reduced fat mass and restored glucose homeostasis. A related concept is exploited in livestock production, where muscle growth‐stimulating agents, such as β_2_‐agonists, are used to increase meat or muscle mass whilst reducing fat mass, a practice known as ‘repartitioning’ (Sillence, 2004). Consistent with this, antibody‐based stimulation of global muscle hypertrophy in humans via myostatin receptor blockade not only increased lean mass by 1.70 kg [80% confidence interval (CI): 1.14 to 2.26 kg] but also reduced fat mass by 7.49 kg (80% CI: −8.33 to −6.64 kg) and lowered glycosylated haemoglobin by 0.76% (80% CI: −1.05% to −0.48%; absolute percentage points) in overweight and obese patients with type 2 diabetes (Heymsfield et al., 2021).

Despite these benefits, the mechanisms by which skeletal muscle hypertrophy improves metabolic health remain poorly understood. One emerging concept is that a hypertrophying skeletal muscle acts as powerful metabolic sink. Skeletal muscle constitutes, on average, 30%–40% of total body mass and, when considered as a single organ, constitutes the largest organ in the human body by both volume and mass (Janssen et al., 2000). In order to expand, skeletal muscle requires a substantial net influx of substrates. For a 75 kg individual, a 1% increase in muscle mass necessitates the accretion of ∼80 g of dry biomass (Forsberg et al., 1991). Although this accretion occurs over weeks, the cumulative demand for amino acids, lipids and carbon precursors represents a significant diversion of nutrients that might otherwise be stored in adipose tissue or contribute to systemic metabolic stress. This persistent ‘nutrient pull’ by the remodelling musculature might effectively repartition substrates away from white adipose tissue, thereby contributing to improvements in metabolic health (Guo et al., 2009; Havers et al., 2025).

A related hypothesis is that hypertrophying muscle selectively consumes circulating metabolic risk factors. The branched‐chain amino acids leucine, isoleucine and valine are not only substrates for muscle protein synthesis (Hosios et al., 2016), but also associated with diabetes, cardiovascular disease and cancer risk, with some evidence for causation (Bhattacharya et al., 2014; Mayers et al., 2014; Murashige et al., 2022; Wang et al., 2011). Consistent with increased branched‐chain amino acid uptake, we previously found that bodybuilders had lower fasting circulating branched‐chain amino acid concentrations than untrained individuals (Schranner et al., 2021).

However, muscle hypertrophy cannot be reduced to protein synthesis alone. The transition from a metabolic steady state to cellular growth necessitates extensive metabolic reprogramming, nucleotide biosynthesis for transfer RNA, mRNA and ribosomal biogenesis, DNA replication and the synthesis of non‐essential amino acids from central carbon intermediates (Baumert et al., 2024; Wackerhage et al., 2022). During such growth‐associated metabolic rewiring, first described by Otto Warburg et al. (1927) and extensively characterized in cancer and proliferative systems, nutrient flux is redirected more towards biomass generation (DeBerardinis & Chandel, 2016; Ducker & Rabinowitz, 2017; Lunt & Vander Heiden, 2011; Peng‐Winkler & Fendt, 2026; TeSlaa et al., 2023; Vander Heiden et al., 2009).

Cellular growth therefore requires coordinated reprogramming not only of intracellular metabolism but also of metabolite uptake and release. in vitro, these exchanges can be quantified by comparing metabolite concentrations in fresh and spent media (Jain et al., 2012). in vivo, arteriovenous metabolite concentration differences, an approach with historical roots in arteriovenous tumour metabolism research (Warburg et al., 1927), enable direct measurement of tissue‐level metabolite exchange. Advances in metabolomics now permit these fluxes to be assessed on a metabolome‐wide scale (Ivanisevic et al., 2015; Schrimpe‐Rutledge et al., 2016), revealing organ‐specific substrate exchange in contexts such as human heart failure (Murashige et al., 2020). Nevertheless, metabolome‐wide uptake and release by hypertrophying skeletal muscle have not been characterized systematically to date.

To address this gap, we designed two complementary experiments. Rather than treating these as directly analogous systems, the in vitro component was designed to isolate the cell‐autonomous anabolic metabolic programme in controlled conditions, whereas the in vivo component captures the integrated physiological response under systemic constraints. Together, they address the following research questions:

1. What is the effect of insulin‐like growth factor‐1 (IGF‐1)‐induced muscle hypertrophy on metabolite uptake and release in differentiated C2C12 myotubes, compared with untreated controls, as assessed by fresh–spent media metabolomics?
2. What is the effect of unilateral leg resistance exercise on metabolite uptake and release in men and women 24 h after exercise, compared with the contralateral non‐exercised leg, as assessed by femoral arteriovenous metabolomics?

## 2 MATERIALS AND METHODS

### 2.1 Ethical approval

The experiments on human participants reported in this study were approved by the local ethics committee of the Technical University of Munich (reference number 2023‐232‐S‐KH) and were conducted in accordance with the *Declaration of Helsinki*, except for registration in a database, because the study constituted a mechanistic, acute exercise‐physiology investigation rather than a clinical trial. Each participant received detailed written and verbal information regarding the study procedures and potential risks and provided written informed consent before enrolment.

### 2.2 in vitro model of muscle anabolism (C2C12 myotubes)

#### 2.2.1 Cell culture and differentiation

C2C12 mouse myoblast cells (ATCC CRL‐1772), kindly provided by Professor Maria Spletter, were cultured in growth medium consisting of high‐glucose Dulbecco's modified Eagle's medium (DMEM; Gibco, 11995, Waltham, MA, USA) supplemented with 10% fetal bovine serum (Biowest, S181B, Nuaillé, France). Unless stated otherwise, 12 × 10^3^ cells were seeded per well in 12‐well cell culture plates, resulting in 70%–80% confluency after 72 h. Myotube differentiation was induced by replacing growth medium with differentiation medium (DMEM supplemented with 2% horse serum; HyClone, 10407223, Marlborough, MA, USA). Differentiation medium was refreshed daily, and myotubes were maintained for 6–7 days. Cells were cultured in a humidified incubator at 37°C with 5% CO_2_ throughout all experiments. Unless otherwise stated, experiments were performed on three independent days, with six replicate wells per condition per experiment (*n* = 18 per medium condition). Each well was treated as an independent biological replicate. Cell passages were limited to a maximum of nine.

#### 2.2.2 IGF‐1 cell treatment

To induce a hypertrophic stimulus, fully differentiated myotubes were treated with insulin‐like growth factor‐1 (IGF‐1; 100 ng/mL, Human IGF‐I Recombinant Protein, PeproTech, 100‐11‐1MG) for 24 h. IGF‐1 stock solutions were prepared in sterile PBS containing 0.1% bovine serum albumin and sterile‐filtered to minimize adsorption to tube surfaces. Control cells received vehicle treatment only.

#### 2.2.3 Myotube diameter analysis

**FIGURE 1:**
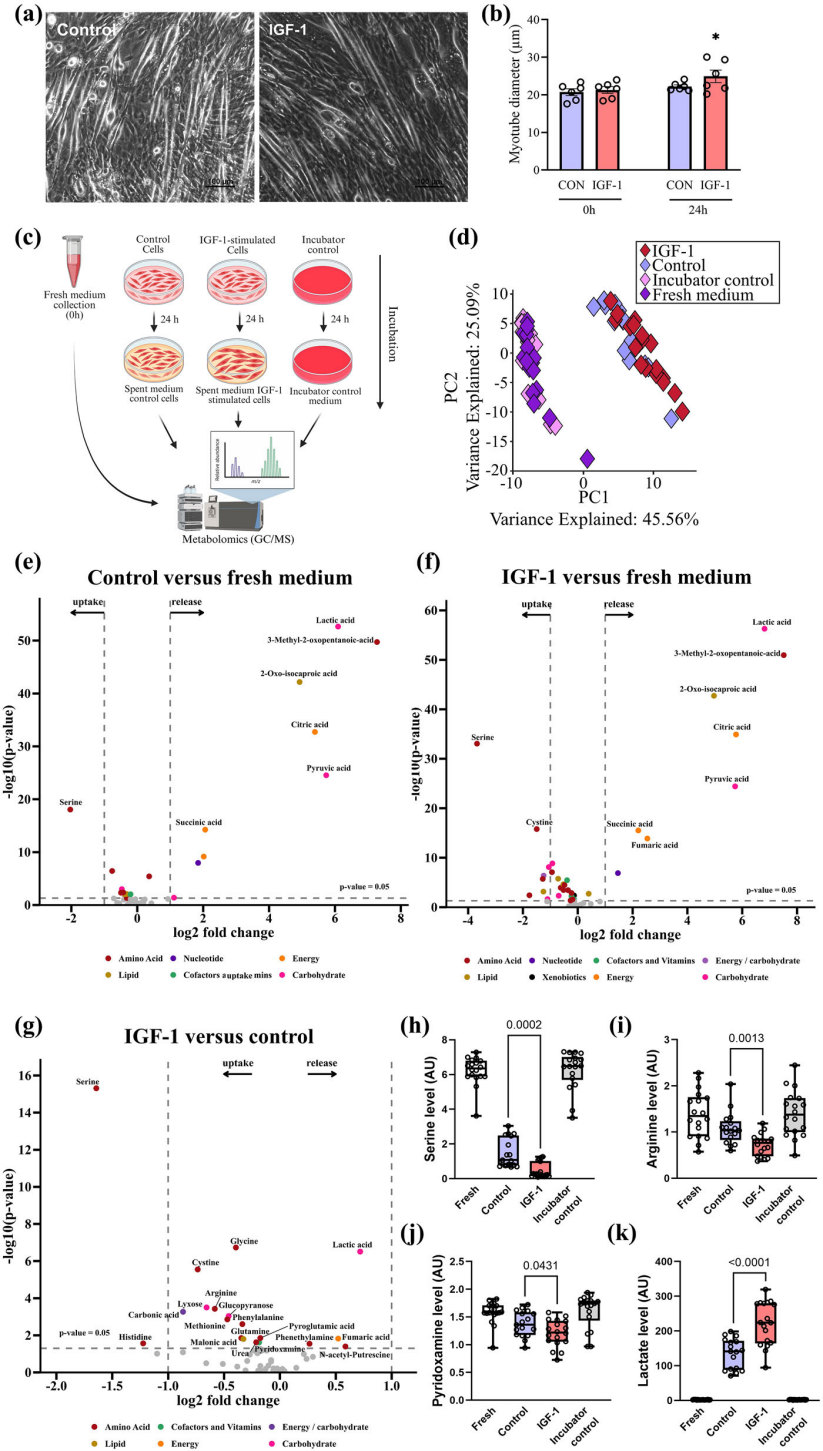
Insulin‐like growth factor‐1 (IGF‐1) stimulation induces myotube hypertrophy and remodels extracellular metabolite exchange in C2C12 myotubes in vitro. (a) Representative brightfield images of C2C12 myotubes following 24 h of vehicle or IGF‐1 treatment. Scale bar: 100 µm. (b) Quantification of myotube diameter immediately before (0 h) and after 24 h of vehicle or IGF‐1 treatment. Data are presented as the mean ± SEM from six independent experiments (*n* = 6), assessed by a two‐way repeated‐measures ANOVA with Šídák's multiple comparisons test. *P < 0.05. (c) Overview of the fresh‐versus‐spent medium metabolomics experimental design. Metabolite concentrations were measured in fresh medium, incubator control medium and spent media from control and IGF‐1‐treated myotubes. (d) Principal component analysis (PCA) of log‐transformed metabolite concentrations measured across the different media conditions, illustrating distinct extracellular metabolomic profiles following cellular conditioning and IGF‐1 stimulation. (e–g) Volcano plots illustrating fold changes (log_2_) and statistical significance of metabolite uptake and release across the different media conditions. Comparisons depict spent medium from control myotubes versus fresh medium (e), spent medium from IGF‐1‐treated myotubes versus fresh medium (f), and spent medium from IGF‐1‐treated versus control myotubes (g). Negative fold changes indicate net uptake; positive fold changes show net release. (h–k) Box plots showing relative extracellular metabolite levels: serine (h), arginine (i), pyridoxamine (j), and lactate (k). Data are shown for fresh medium, incubator control medium, and spent media from control and IGF‐1‐treated myotubes. Each data point represents an individual well. These metabolite levels illustrate the metabolite changes underlying the fold‐change analyses shown in panels. Figure 1c was created with BioRender.

To assess the hypertrophic response to IGF‐1 treatment, myotube diameters were measured immediately before (0 h) and after 24 h of IGF‐1 or vehicle treatment. Six independent experiments performed on different days were included in this analysis (biological replicates, *n* = 6). At least three brightfield images per well were acquired at ×10 magnification using an AxioCam Mrc5 camera mounted on a Carl Zeiss microscope. Diameters were measured transversally in a minimum of 10 myotubes per image at three locations along the length of each myotube using ImageJ software (National Institute of Health, Bethesda, MD, USA), taking into account the pixel‐to‐aspect ratio. Consequently, a minimum of 30 myotubes were analysed per well. The same wells were imaged at both time points, allowing paired analysis of changes in myotube diameter over time (Figure 1a).

#### 2.2.4 Fresh versus spent media sampling

To assess cellular metabolite consumption and release, culture medium samples were collected immediately before (fresh) and after (spent) 24 h of cell treatment, as previously described (Jain et al., 2012). Samples were subsequently subjected to targeted metabolite profiling by gas chromatography–mass spectrometry. Cell‐free medium controls were incubated in parallel in identical conditions, without cell contact for the duration of the treatment period to account for non‐cell‐dependent changes, including spontaneous degradation, evaporation and adsorption to cultureware.

#### 2.2.5 Metabolomics

Cell media were processed for metabolomics as described before (Sapcariu et al., 2014). Metabolites were extracted from 5 µL of cell culture medium by adding 45 µL of ice‐cold methanol/water (8:1, v/v) containing D_6_‐glutaric acid as an internal standard. Samples were mixed at 1400 rpm for 10 min at 4°C and centrifuged at 13 000*g* for 10 min at 4°C. Forty microlitres of the resulting supernatant were transferred to glass vials, evaporated to dryness under vacuum at 4°C (Labconco CentriVap), and stored at 4°C until analysis. For gas chromatography–mass spectrometry measurement, dried metabolites were derivatized using an automated Gerstel MPS sampler: samples were incubated with 15 µL of 2% methoxyamine hydrochloride in pyridine at 55°C for 90 min, followed by addition of 15 µL N‐methyl‐N‐(tert‐butyldimethylsilyl)trifluoroacetamide (MTBSTFA) and further incubation for 60 min at 55°C. One microlitre of the derivatized sample was injected in splitless mode at 270°C into an Agilent 7890B gas chromatograph equipped with a DB‐35 ms capillary column (30 m + 5 m Duraguard). Helium served as the carrier gas at 1.0 mL/min. The gas chromatograph oven was held at 100°C for 2 min, then ramped at 10°C/min to 300°C. Detection was performed using an Agilent 5977B mass spectrometer under electron ionization (70 eV), with source and quadrupole temperatures of 230°C and 150°C, respectively, operating in selected‐ion monitoring mode. Peak identification, quantification and natural‐abundance correction of mass isotopomer distributions were conducted using MetaboliteDetector software. D_6_‐glutaric acid was used for signal normalization across samples.

#### 2.2.6 Statistical analysis

Myotube diameter data were analysed in GraphPad Prism (v.10.3.1) using a two‐way repeated‐measures ANOVA, with treatment (vehicle vs. IGF‐1) and time (0 h vs. 24 h) as factors. Šídák's multiple comparisons test was used for *post hoc* analyses. Statistical significance was defined as *P* < 0.05.

Metabolite abundances were log‐transformed for statistical analysis. To quantify cellular uptake and release, we calculated the log fold change (logFC) for each metabolite as the difference between spent and fresh medium: logFC = log(*M*_spent_) − log(*M*_fresh_), where *M* denotes the normalized metabolite abundance. A negative fold change indicates net cellular uptake, whereas a positive fold change indicates net release. To quantify the metabolic effect of IGF‐1, we additionally calculated the logFC between experimental groups as the difference in log‐transformed metabolite abundances. In this context, a negative logFC indicates that IGF‐1 stimulation increased metabolite uptake (or reduced the release) relative to control cells, whereas a positive logFC indicates increased release (or reduced uptake). To account for the hierarchical structure of the data and potential batch effects, differences in metabolite abundances between experimental conditions were assessed using a linear mixed‐effects model. Experimental condition was treated as a fixed effect, and experimental day (corresponding to the three independent experiments) was included as a random effect.

Where appropriate, *post hoc* comparisons were performed using Fisher's least significant difference (LSD) procedure, implemented in MATLAB (R2025a) via the *multcompare* function. The *P*‐values were adjusted for multiple comparisons using the Benjamini–Hochberg false discovery rate (FDR) procedure. Statistical significance was defined as FDR‐adjusted *P* < 0.05. Nominal *P*‐values are provided in Supporting Information (Supplement 1). Data visualization was performed using R (v.4.4.1, *ggplot2*, *tidyverse*) and GraphPad Prism (v.10.3.1).

### 2.3 in vivo human arteriovenous experiment

#### 2.3.1 Participants

Eight healthy, resistance‐untrained participants were originally recruited; however, one participant was excluded from the final metabolomics analysis owing to incomplete data acquisition. Consequently, the final analytical cohort consisted of seven participants (three males, four females; age 25.6 ± 3.2 years; body mass 71.9 ± 12.3 kg; height 173.6 ± 7.8 cm; body mass index 23.8 ± 2.8 kg/m^2^). All participants received a hypertrophic stimulus via a lower‐body resistance training intervention. We aimed to investigate the effect of the hypertrophic response on the arterial and venous blood metabolome, analogous to the fresh versus spent media experiment performed in vitro. Participants were free of any neoplastic, cardiovascular, pulmonary or metabolic disorders. All volunteers were recruited from the local Munich area and screened by a licensed physician prior to participation.

#### 2.3.2 Resistance training protocol

Participants completed three preparatory lower‐body resistance training sessions 1–2 weeks before the experimental trial to minimize delayed‐onset muscle soreness and ensure technical proficiency. Each session included unilateral leg‐extension, leg‐curl and calf‐raise exercises. To establish balanced neuromuscular adaptations during the initial habituation, the first two sessions were performed unilaterally on both legs in an alternating fashion (two and three sets per exercise, respectively). From the third preparatory session onwards, the study moved to a randomized unilateral design: one leg was randomly designated as the trained limb, while the contralateral leg served as the non‐exercise control. This final preparatory session (four sets per exercise) and the subsequent experimental trial (five sets per exercise) were performed to concentric muscle failure with the trained leg only. All sessions were conducted at the Technical University of Munich between 11:00 and 13:00 h.

Each session began with a 5 min stationary bike warm‐up followed by two exercise‐specific sets. Loads were adjusted between sessions and sets to maintain a target range of 8–12 repetitions; weights were increased if >12 repetitions were completed and reduced if <8 repetitions were achieved. A 3 min rest period was provided between all sets and exercises, and participants rested for ≥48 h between preparatory sessions.

**FIGURE 2:**
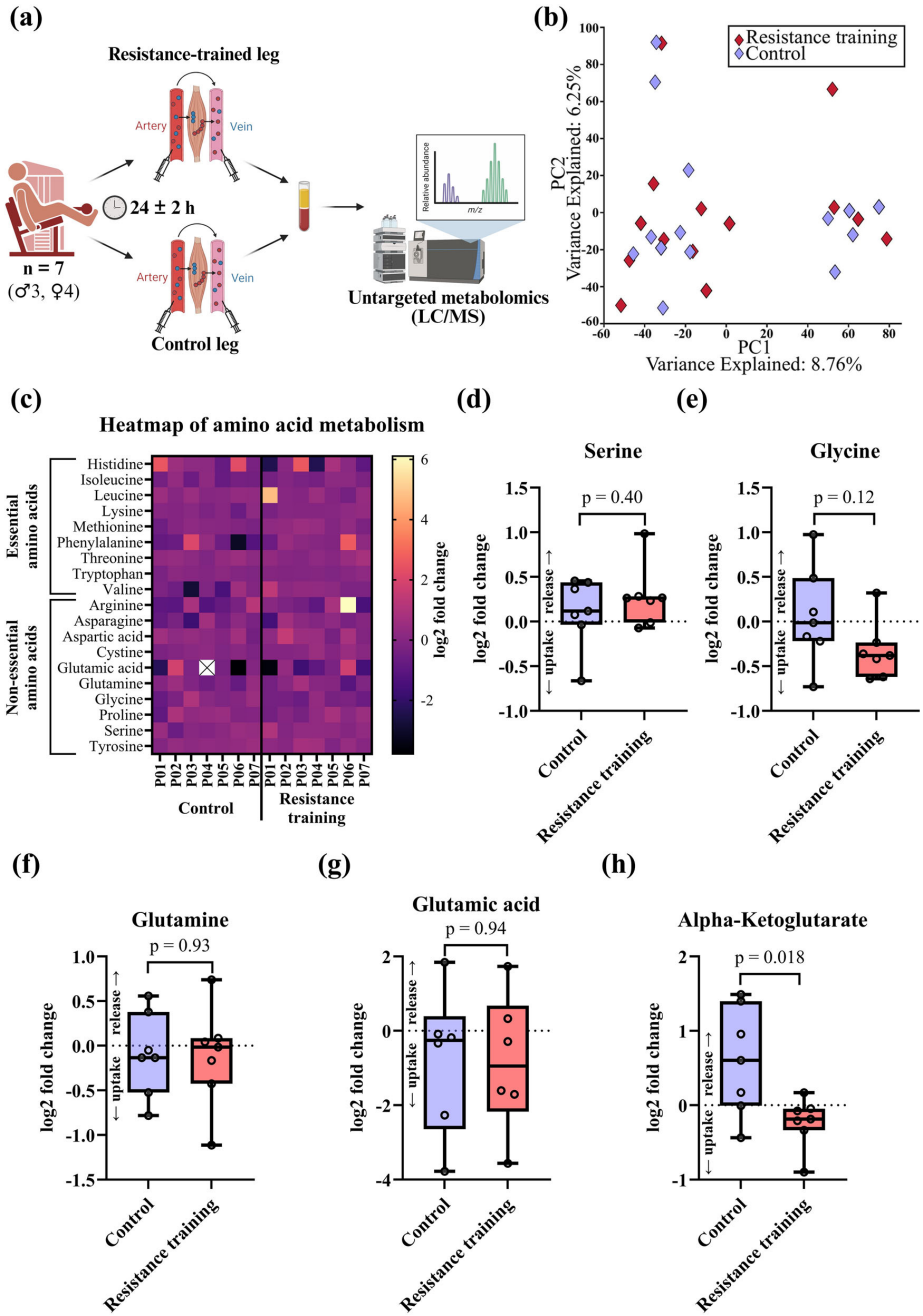
Study design, amino acid uptake and release, and specific metabolic pathways. (a) Study design. Participants completed three familiarization resistance training sessions, followed by a one‐legged experimental training session. One leg served as the control leg. Approximately 24 h later, blood samples were collected from the femoral artery and vein of both legs. Blood samples were processed to plasma and analysed using untargeted metabolomics. (b) Principal component analysis of plasma metabolite profiles from the resistance‐trained leg and control leg. (c) Heatmap of systemic amino acid modulation. The heatmap displays the log2 fold change of essential and non‐essential amino acids per subject. Colours indicate net uptake (negative values, dark) or net release (positive values, bright) by the respective leg. X = missing data. Each square represents the metabolic modulation of the corresponding amino acid in one subject. (d, e) Boxplots illustrating one‐carbon metabolism. Net uptake or release fold changes for serine (d) and glycine (e). Data presented as boxplots. (f–h) Box plots illustrating the glutamine (f), glutamic acid (g) and α‐ketoglutaric acid (h) metabolic pathway (without false discovery rate correction). Each data point represents an individual participant. Figure 2a was created with BioRender.

We ran the experimental trial ≥72 h after familiarization (Figure 2a). During this trial, the trained leg performed five sets per exercise to concentric failure. We collected arteriovenous blood samples ∼24 ± 2 h post‐session, a time point assumed to show peak postexercise protein synthesis relative to baseline (Miller et al., 2005). Participants maintained a standardized low‐protein meal plan and avoided strenuous activity for 24 h prior to sampling, with the final meal consumed ≥4 h before the procedure to ensure a postabsorptive state (for the nutritional plan, see Supporting Information, Supplement 2).

#### 2.3.3 Arteriovenous blood sampling

Arterial and venous blood samples were collected from the femoral artery and vein of both legs. Samples were drawn into EDTA tubes, gently inverted, and immediately centrifuged at room temperature (2500*g*, 10 min). Plasma was pipetted into a new microfuge tube, gently mixed with the pipette and aliquoted into cryogenic tubes (Sarstedt AG & Co. KG, Nümbrecht, Germany). The aliquots were immediately placed on dry ice and stored at −80°C until further analysis. Samples were then transported on dry ice to the Metabolomics and Proteomics Core Facility at Helmholtz Centre Munich for metabolomic profiling. All samples were pseudoanonymized and analysed in randomized order.

#### 2.3.4 Non‐targeted plasma metabolomics

Plasma samples were analysed using liquid chromatography–high resolution mass spectrometry (LC‐HRMS)‐based non‐targeted metabolomics. All chemicals were of analytical grade or higher, and we used LC–MS‐grade solvents for all LC–MS analyses.

Plasma samples were extracted as previously described (Artati et al., 2025). Briefly, we thawed frozen plasma on ice and prepared pooled quality control samples by combining aliquots from each sample. A commercial plasma sample served as a long‐term reference. For extraction, 100 µL of plasma or quality control sample was mixed with 500 µL of extraction solvent (methanol containing internal standards) and shaken at 1200 rpm for 2 min. Following centrifugation at 2650 rpm, 50 µL of the supernatant was transferred into each of four 96‐well plates, followed by solvent evaporation under nitrogen using a TurboVap 96 system (Biotage). The plates were then sealed and the dried extracts stored at −80°C until analysis.

For reversed‐phase (RP) separation, we performed LC‐HRMS/MS measurements on a Sciex ExionAD LC system coupled to a Sciex ZenoTOF 7600 tandem mass spectrometer equipped with a Turbo V/OptiFlow Turbo V electrospray ionization (ESI) source. Before analysis, two aliquots of each dried extract were reconstituted in LC‐compatible solvents containing chemical standards at fixed concentrations. One aliquot was analysed in positive ionization mode and the other in negative ionization mode using separate injections. Metabolite separation utilized a Phenomenex Kinetex C18 column (100 mm × 2.1 mm, 1.7 µm particle size) with a linear gradient from eluent A (100% water with 0.1% formic acid) to eluent B (100% acetonitrile with 0.1% formic acid). The flow rate was set to 0.5 mL/min, column temperature to 40°C, and injection volume to 5 µL. Detailed LC and MS parameters are reported by Artati et al. (2025).

For hydrophilic interaction liquid chromatography (HILIC) separation, we performed LC‐HRMS/MS measurements using an Agilent 1290 Infinity II BioLC system coupled to the same Sciex ZenoTOF 7600 mass spectrometer. Dried extracts were reconstituted as described above, with one aliquot analysed in positive ionization mode. Separation was achieved on an Agilent Infinity Poroshell 120 HILIC‐Z column (100 × 2.1 mm, 2.7 µm particle size, PEEK‐lined) using a gradient from eluent A (100% water with 10 mM ammonium formate and 0.1% formic acid) to eluent B (10% water/90% acetonitrile with 10 mM ammonium formate and 0.1% formic acid). Flow rate, column temperature and injection volume remained consistent with the RP method. Detailed LC and MS parameters for both methods are provided by Artati et al. (2025).

Initial data quality control involved checking the retention time, *m/z* and intensity of internal standards, all of which were detected with a mass error of ≤5 ppm. Data processing in MZmine 4.5 included recalibration, peak detection and alignment, gap filling, isotope grouping and metabolite annotation (Heuckeroth et al., 2024). Metabolites were annotated by matching features against an in‐house spectral library and multiple external databases and by using the in silico tools SIRIUS and CSI:FingerID with high COSMIC confidence scores (Dührkop et al., 2015, 2019; Hoffmann et al., 2022). We reported annotation confidence according to the Metabolomics Standards Initiative and the classification proposed by Schymanski et al. (Schymanski et al., 2014; Sumner et al., 2007). Finally, processed data were exported from MZmine as .csv files for downstream analysis in R and Excel, retaining only features present in all quality control samples with a relative SD of <30%.

#### 2.3.5 Statistical quantification of metabolic exchange

We performed all statistical analysis using MATLAB (R2025a). Metabolomics data were log‐transformed for statistical analysis and were applied identically to arterial and venous samples from both trained and untrained legs. Leg‐specific venous‐to‐arterial log fold changes (logFC) were calculated as an indicator of metabolic directionality across the sampled limb circulation: logFC = log(Mv) − log(Ma), where Mv denotes the metabolite abundance in venous blood and Ma denotes the metabolite abundance in arterial blood. Net uptake or release was inferred from these venous‐to‐arterial differences in relative metabolite abundance, reflecting metabolic directionality rather than quantitative flux. A negative logFC indicates net uptake by skeletal muscle (venous < arterial), whereas a positive logFC indicates net release into the circulation (venous > arterial).

Significant net uptake or release of individual metabolites within each leg was assessed using Student's paired *t*‐tests comparing arterial and venous samples across participants. To evaluate whether resistance training altered metabolite exchange, logFC values were compared between the trained and untrained legs using Student's paired *t*‐tests. Statistical significance was defined as *P* < 0.05. To account for multiple testing across metabolites, *P*‐values were adjusted using the Benjamini–Hochberg FDR procedure. All figures were generated using R (v.4.4.1, *ggplot2*, *tidyverse*) or GraphPad Prism (v.10.3.1).

## 3 RESULTS

### 3.1 IGF‐1 increases myotube diameter in differentiated C2C12 myotubes

To verify that IGF‐1 exerted an anabolic effect in our experimental conditions, we quantified myotube diameters immediately before (0 h) and after 24 h of treatment. Although baseline diameters were similar between groups (20.7 ± 0.9 µm for controls vs. 21.3 ± 0.9 µm for IGF‐1), IGF‐1 treatment increased myotube diameter by 3.6 ± 0.9 µm over 24 h (*P* = 0.005), compared with an increase of 1.5 ± 0.5 µm in vehicle‐treated controls (Figure 1a, b).

### 3.2 C2C12 fresh versus spent media

To find out how stimulation of muscle hypertrophy affects metabolite uptake and release in vitro, we compared the metabolomes of fresh and spent culture media from differentiated C2C12 myotubes treated with IGF‐1 or vehicle control (Figure 1c). We detected 58 annotated metabolites, including 23 metabolites associated with amino acid metabolism, 18 metabolites involved in carbohydrate and energy metabolism, 8 lipid‐related metabolites, 4 cofactors and vitamins, 1 nucleotide and 4 xenobiotic or unknown metabolites.

Principal component analysis of log‐transformed metabolite concentrations revealed a separation between fresh medium, cell‐free controls and spent media, suggesting systematic changes in metabolite uptake and release between control and IGF‐1‐stimulated C2C12 myotubes (Figure 1d). Spent media from control cells showed a shift relative to baseline medium. In contrast, media from IGF‐1‐treated myotubes exhibited the greatest displacement along the primary principal components (PCs). Compared with analyses of untransformed data (Supporting Information, Supplement 1), group separation was attenuated after log transformation, reflecting reduced dominance of high‐abundance metabolites. A PCA of untransformed data is provided to illustrate separation driven by absolute concentration differences (Supporting Information, Supplement 1).

Fold‐change analysis of spent relative to fresh media demonstrated condition‐specific patterns of cellular metabolite uptake and release (Figure 1e–g). Control myotubes displayed comparatively modest net metabolite exchanges, whereas IGF‐1 stimulation enhanced uptake of multiple amino acids and other metabolites involved in biosynthesis. Notably, IGF‐1‐treated myotubes significantly took up more serine (IGF‐1 vs. control: FC −1.64, *P* < 0.001, FDR < 0.001), arginine (FC −0.58, *P* < 0.01, FDR = 0.004) and pyridoxamine (FC −0.19, *P* = 0.024, FDR n.s.) when compared with control myotubes (Figure 1e–g). These metabolite uptake patterns are reflected in the corresponding raw metabolite concentrations in fresh and spent media, in which IGF‐1‐treated cells exhibited lower extracellular levels of serine, arginine and pyridoxamine than controls (Figure 1h–k). Additional uptake of metabolites linked to central carbon metabolism and cofactor availability was observed upon IGF‐1 stimulation (see Supporting Information, Supplement 1). In parallel, IGF‐1 stimulation increased lactate release into the culture medium (FC +0.72, *P* < 0.001, FDR < 0.001; Figure 1e–g, k).

Together, these data indicate that IGF‐1‐stimulated myotubes take up nitrogen‐ and cofactor‐related substrates whilst releasing lactate, suggesting a Warburg effect‐like reprogramming of metabolite uptake and release during skeletal muscle hypertrophy.

### 3.3 Human arteriovenous metabolomics

To investigate the effect of skeletal muscle hypertrophy stimulation in vivo, participants performed one‐legged resistance exercise, with the contralateral leg serving as a non‐exercise control. After 24 h, we obtained blood samples from the femoral artery and vein of the exercise and control legs, processed the blood to plasma and analysed it by LC‐HRMS (Figure 2a). Our untargeted analysis identified 295 annotated metabolites (Supporting Information, Supplement 2). Among these, 123 metabolites were associated with lipid metabolism, 82 with amino acid metabolism, 39 with peptide metabolism, 8 with cofactor and vitamin metabolism, 6 with nucleotide metabolism, 2 with energy metabolism and 35 with xenobiotics and unknown metabolites.

Importantly, although nominal differences (*P* < 0.05) were identified for individual metabolites, none survived correction for multiple testing using the Benjamini–Hochberg FDR procedure. The following results therefore describe nominally significant patterns and directional trends, which should be interpreted as hypothesis‐generating rather than confirmatory findings.

### 3.4 Is amino acid uptake and release altered by resistance training?

Given that resistance training can cause a ∼3‐fold increase in muscle protein synthesis 24 h after exercise (Miller et al., 2005), we hypothesised a large increase of all amino acids in the resistance‐trained leg. Surprisingly, our PCA of 295 metabolites revealed only subtle differences in global metabolite uptake and release between the exercised and control legs 24 h after resistance training (Figure 2b). PC1 and PC2 accounted for 8.76% and 6.25% of the total variance, respectively, indicating a small but discernible shift in metabolite uptake and release.

Contrary to our expectation, we observed no statistically significant differences in the net uptake or release of essential or semi‐essential/non‐essential amino acids between the resistance‐trained and control legs. The responses also varied across subjects (Figure 2c). Likewise, we observed no preferential net uptake or release in key amino acids related to one‐carbon metabolism, specifically serine (Figure 2d) and glycine (Figure 2e). However, upon examination of the glutamine–glutamic acid–α‐ketoglutaric acid pathway (see Figure 2f–h), we observed a nominally significant uptake of α‐ketoglutaric acid (*P* = 0.018, FDR = 0.946) in the resistance‐trained leg (Figure 2h), which might suggest a sustained anaplerotic demand to replenish the tricarboxylic acid cycle and support anabolic signalling, although this interpretation warrants cautious consideration given the absence of FDR‐corrected significance.

### 3.5 Does the stimulation of muscle hypertrophy by resistance training alter the uptake and release of metabolites, including metabolic risk factors?

**FIGURE 3:**
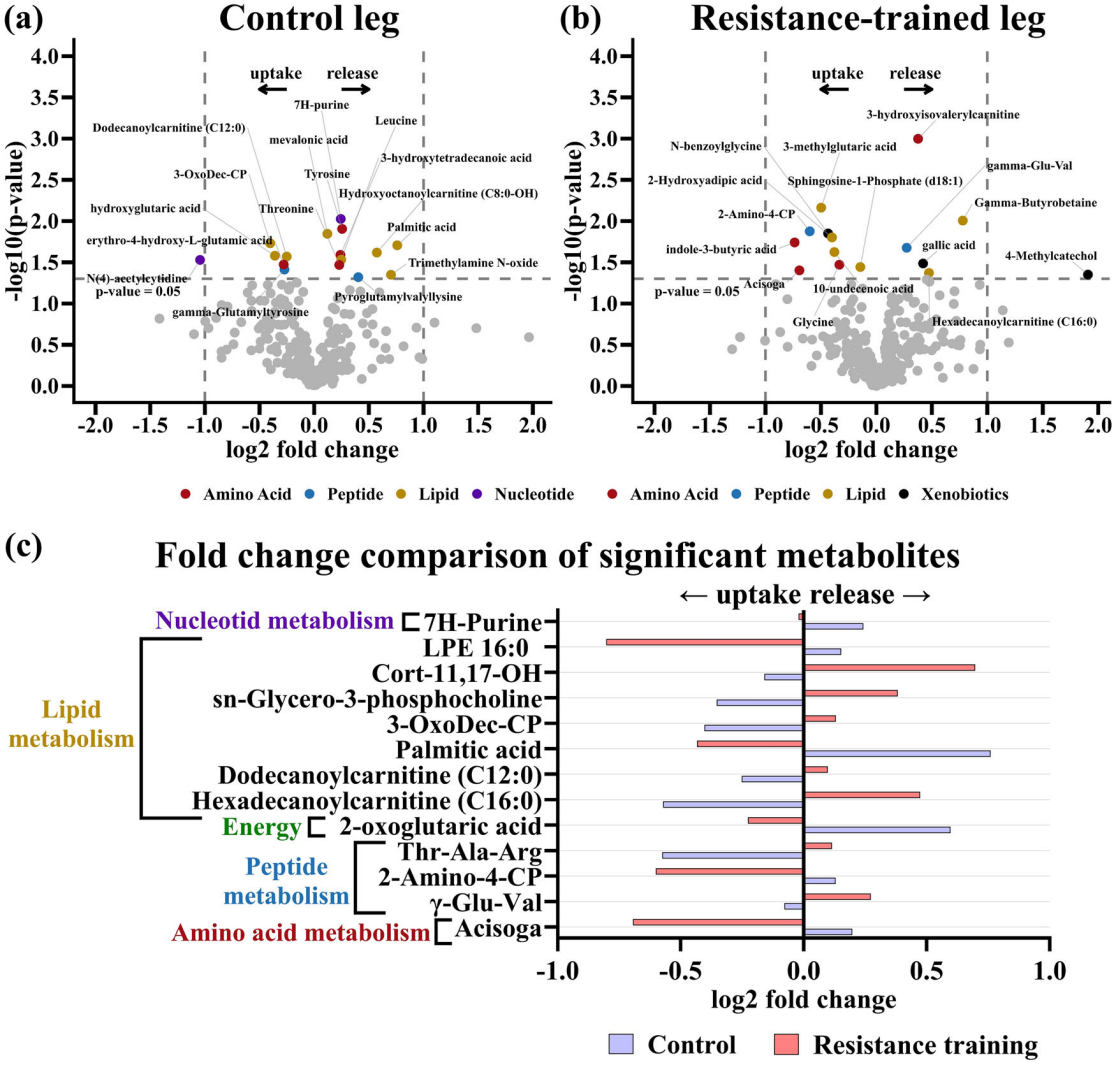
Uptake and release of metabolites in response to resistance training. (a, b) Volcano plots displaying the uptake and release of metabolites ∼24 h after resistance training in the control (a) and resistance‐trained (b) legs. Each data point corresponds to a specific metabolite, with the log_2_(fold change) value plotted against the −log_10_(*P*‐value) metric. Significant metabolites (*P* < 0.05, without false discovery rate correction) are colour‐coded according to their associated metabolic pathways. (c) Fold change comparison of significant metabolites in control and resistance‐trained legs (*P* < 0.05, without false discovery rate correction). Abbreviations: Cort‐11,17‐OH, (8S,9S,10R,11S,13S,14S,17R)‐11,17‐dihydroxy‐17‐(2‐hydroxyacetyl)‐10,13‐dimethyl‐decahydro‐1*H*‐cyclopenta[a]phenanthren‐3‐one or similar isomer (putative level 2 annotation); LPE 16:0, lysophosphatidylethanolamine 16:0 (putative level 3 annotation); 2‐Amino‐4‐CP, 2‐amino‐4‐{[2‐(carbamoylamino)‐1‐carboxyethyl]carbamoyl}butanoic acid putative level 3 annotation); 3‐OxoDec‐CP, 3‐[(3‐oxodecan‐2‐yl)carbamoyl]propanoic acid.

We next assessed net metabolite uptake (negative fold change) and release (positive fold change) within each leg. The control leg showed differential abundance of 16 metabolites (Figure 3a), whereas the trained leg modulated 15 metabolites (Figure 3b). Notably, the trained leg took up several metabolites related to amino acid and peptide metabolism, including glycine, indole‐3‐butyric acid and *N*‐(3‐acetamidopropyl)pyrrolidin‐2‐one (acisoga). In contrast, it released metabolites involved in carnitine metabolism and fatty acid transport, such as γ‐butyrobetaine and hexadecanoylcarnitine (C16:0). However, after correcting for multiple testing using FDR, none of the individual metabolites remained statistically significant.

A direct comparison of arteriovenous differences revealed 15 metabolites that differed nominally between the trained and control legs (all *P* < 0.05, none surviving FDR correction; see Figure 3c for 13 of these metabolites; two xenobiotic metabolites are listed in Supporting Information, Supplement 2). The trained leg took up more acisoga and 2‐amino‐4‐CP (putative level 3 annotation; for full metabolite name, see Figure 3 legend), while releasing γ‐Glu‐Val and Thr‐Ala‐Arg (putative level 3 annotation). Lipid metabolism showed distinct divergence. Specifically, the resistance‐trained leg released C12:0 and C16:0 carnitines, Cort‐11,17‐OH (putative level 2 annotation; for full metabolite name, see Figure 3 legend) and sn‐glycero‐3‐phosphocholine (putative level 2 annotation), all of which were taken up by the control leg. Conversely, the resistance‐trained leg took up LPE 16:0 (putative level 3 annotation) and palmitic acid (hexadecanoic acid), in addition to the energy/cofactor metabolite α‐ketoglutaric acid, which was released in the control leg instead. Together, these nominal findings suggest that resistance‐trained muscles might adopt a distinct metabolite exchange pattern 24 h postexercise, involving preferential uptake of energy substrates and divergent handling of lipid species compared with resting muscle.

## 4 DISCUSSION

The main finding of this study was that the stimulation of muscle hypertrophy in vitro by IGF‐1 was associated with an increased uptake of amino acids, including serine, and enhanced lactate secretion, indicative of a Warburg‐like metabolic reprogramming. In contrast, and contrary to expectation, resistance‐trained human legs studied in the postabsorptive state did not show increased amino acid uptake 24 h after resistance training, and overall differences in metabolite uptake and release compared with the contralateral control leg were modest.

### 4.1 Anabolic reprogramming in IGF‐1‐stimulated myotubes

IGF‐1 stimulation induced a coordinated anabolic metabolic programme consistent with skeletal muscle growth. IGF‐1‐treated myotubes increased uptake of serine and glycine and showed enhanced arginine uptake and elevated lactate release, indicating a shift in extracellular substrate exchange towards anabolism.

The increase in serine uptake highlights the central role of serine metabolism in supporting muscle growth, because serine metabolism links glycolytic carbon flux to one‐carbon metabolism required for nucleotide synthesis, redox homeostasis and biomass production (Mäntyselkä et al., 2024). In rapidly proliferating cancer cells, metabolite exchange profiling similarly identified glycine and serine metabolism as critical determinants of growth, with increased glycine consumption distinguishing highly proliferative phenotypes (Jain et al., 2012). Our findings suggest that hypertrophying myotubes engage a metabolic programme partly overlapping with proliferating cells, characterized by increased demand for serine and glycine to sustain anabolic processes. In line with this, the serine synthesis pathway enzyme Phosphoglycerate dehydrogenase (PHGDH) has been identified as rate limiting for myotube hypertrophy, anabolic metabolism, and mammalian target of rapamycin complex 1 (mTORC1) signalling, positioning serine metabolism upstream of growth control in skeletal muscle (Mäntyselkä et al., 2024). The increased extracellular serine uptake observed here is therefore likely to reflect anabolic requirements that exceed endogenous synthesis capacity under IGF‐1 stimulation.

IGF‐1‐treated myotubes also showed increased arginine uptake, consistent with the dual role of arginine as both a proteinogenic amino acid and a direct activator of mTORC1 via lysosomal sensing mechanisms (González & Hall, 2017; Wyant et al., 2017). This finding supports the interpretation that IGF‐1 reinforces mTOR‐dependent anabolic signalling not only through canonical Akt–Tuberous Sclerosis Complex 2 (TSC2) regulation but also through substrate‐driven nutrient sensing.

Concomitantly, IGF‐1‐treated myotubes showed increased lactate release, indicative of a shift towards aerobic glycolysis. Such a Warburg‐like metabolic phenotype has been proposed as a defining feature of hypertrophying muscle in both myotubes and human skeletal muscle (Baumert et al., 2024; Mäntyselkä et al., 2025; Stadhouders et al., 2023; Wackerhage et al., 2022). Rather than implying elevated ATP demand per se, this shift is likely to reflect a reallocation of glycolytic carbon towards biosynthetic intermediates required for cellular growth. The increased uptake of pyridoxamine, a vitamin B_6_ derivative, further suggests elevated cofactor demand to sustain transamination reactions and nitrogen handling in anabolic conditions.

Collectively, our fresh‐versus‐spent media metabolomics approach defines a cell‐autonomous metabolic reprogramming driven by IGF‐1 that prioritizes serine‐dependent one‐carbon metabolism, mTORC1 support and increased glycolytic flux to enable muscle hypertrophy. To our knowledge, this is one of the first applications of extracellular metabolite exchange profiling in differentiated skeletal muscle cells, extending concepts previously established in cancer metabolism to the field of muscle biology.

### 4.2 Systemic substrate availability constrains the local anabolic metabolic signature 24 h after resistance training

In contrast to the IGF‐1‐stimulated myotubes, our human arteriovenous data revealed a subtle and heterogeneous metabolic response 24 h after resistance training. Global metabolomic structure differed only marginally between trained and control legs (PC1 + PC2 < 15% total variance). This suggests that at this specific postexercise time point, whole‐body metabolic homeostasis and the postabsorptive state substantially limit the modulation of large, localized muscle‐specific metabolite exchanges.

Resistance training elevates protein synthesis for a period of ≥48 h (Damas et al., 2015). Notably, Miller et al. (2005) reported a 3‐fold increase in protein synthesis rate 24 h after resistance exercise compared with baseline, with sustained yet diminished rates 48 and 72 h after exercise. Although resistance exercise increases muscle protein synthesis in fasted and fed individuals, the net protein balance, the variable that determines metabolite uptake and exchange, depends on extracellular amino acid availability, as demonstrated in a classic study by the late Kevin Tipton and colleagues (Tipton et al., 1999). Our observation of minimal net amino acid uptake supports the theoretical framework of ‘anabolic potential’ versus ‘substrate availability’; the muscle tissue possesses an augmented capacity for protein synthesis at 24 h (the machinery is primed), but absence of a postprandial nutrient surge appears to prevent a substantial net extraction of building blocks from the circulation. Consequently, the lack of detectable net amino acid exchange is likely to reflect a state in which muscle protein synthesis and breakdown are balanced in the postabsorptive condition, resulting in minimal net substrate retention despite ongoing tissue remodelling.

### 4.3 Sustained demand for tricarboxylic acid cycle anaplerosis and anabolic signalling

Despite the absence of a generalized amino acid uptake, we observed a nominally significant uptake of α‐ketoglutaric acid (*P* = 0.018, FDR = 0.946) in the resistance‐trained leg (FC = −0.22), whereas the contralateral control leg showed net α‐ketoglutaric acid release (FC = 0.59). α‐Ketoglutaric acid connects carbon and nitrogen metabolism, serving as an anaplerotic substrate to fuel the tricarboxylic acid cycle and acting as a precursor for glutamate and glutamine synthesis (Legendre et al., 2020; Naeini et al., 2023). Increased α‐ketoglutaric acid uptake might therefore reflect sustained demand for tricarboxylic acid cycle replenishment and nitrogen handling in the postexercise muscle.

Beyond energy production, α‐ketoglutaric acid emerged as a signalling molecule with anabolic functions. Resistance exercise was shown to elevate intramuscular α‐ketoglutaric acid concentrations in rodent models, consistent with increased uptake or retention (Yuan et al., 2020). In addition, α‐ketoglutaric acid supplementation has been linked to the activation of mTOR signalling in pigs (Chen et al., 2018) and piglets (Wang et al., 2016), whilst suppressing protein degradation and promoting muscle hypertrophy in mice (Cai et al., 2018; Yuan et al., 2020) and enhancing satellite cell proliferation (Xu et al., 2024). Thus, we tentatively propose that the nominal uptake of α‐ketoglutaric acid 24 h postexercise might represent a sustained anabolic signal, although this interpretation requires validation in larger, adequately powered studies.

### 4.4 Skeletal muscle as a metabolic sink for systemic disease risk factors

A central finding of our study is that 24 h after one‐legged resistance training, the hypertrophying muscle might act as a selective ‘sink’ for metabolites linked to cardiovascular and metabolic dysfunction. One such metabolite is *N*‐(3‐acetamidopropyl)pyrrolidin‐2‐one, or acisoga. Although the resistance‐trained leg showed nominally greater uptake of acisoga (*P* = 0.012, FDR = 0.946), we observed a net release in the contralateral control leg. Acisoga is a catabolic product of the polyamine pathway, specifically formed from *N*_1_‐acetylspermidine (Seiler et al., 1982). Systemically, elevated concentrations of acisoga are correlated with reduced left ventricular function (Puetz et al., 2022), higher body mass index (Moore et al., 2018) and type 2 diabetes (Rebholz et al., 2018). Given that polyamines are essential for cell growth (Thomas & Thomas, 2001), our observed acisoga uptake in the resistance‐trained leg might reflect the recycling of polyamine backbones to support muscle remodelling.

Likewise, we observed an increased uptake of palmitic acid (hexadecanoic acid) in the trained leg, whereas the control leg exhibited a net release of palmitic acid. A prospective meta‐analysis has identified palmitic acid as a biomarker for increased type 2 diabetes mellitus risk (relative risk 1.32 [1.18–1.47]; Sun et al., 2020). Although improvements in glucose homeostasis following resistance training are driven primarily by an increase in muscle mass, upregulated GLUT4 expression and enhanced insulin signalling efficiency over time (Dela et al., 2004; Holten et al., 2004; Juel et al., 2004; Wojtaszewski et al., 2005), the observed extraction of palmitic acid by the trained limb represents a distinct metabolic feature. Rather than being a primary driver of glyacemic control, this apparent ‘sink’ function is likely to indicate that hypertrophying muscle prioritizes the clearance of circulating saturated fatty acids to meet its heightened demand for energy or structural components during recovery.

Finally, the exercised leg released more γ‐butyrobetaine. γ‐Butyrobetaine is an intermediate in the l‐carnitine–trimethylamine N‐oxide pathway (Koeth et al., 2019), where trimethylamine N‐oxide is assumed to be a thrombotic and cardiometabolic risk factor (Guasti et al., 2021; Zhu et al., 2016). Notably, the control leg nominally released trimethylamine N‐oxide into the circulation (*P* = 0.044, FDR = 0.877), whereas the trained leg did not. A possible explanation is that resistance training acutely alters the ‘atherogenic’ potential of the venous effluent of the limb by prioritizing local carnitine turnover over systemic trimethylamine N‐oxide synthesis.

It should be noted that several of the differentially exchanged metabolites, including 2‐amino‐4‐CP, Cort‐11,17‐OH and LPE 16:0, carry only putative level 2 or level 3 annotations. The pathway‐level interpretations associated with these features should therefore be considered preliminary until confirmed by targeted liquid chromatography–tandem mass spectrometry with authentic standards.

### 4.5 Lipid turnover and membrane remodelling

Resistance training led to a net release of medium‐ to long‐chain acylcarnitines, including dodecanoylcarnitine (C12:0) and hexadecanoylcarnitine (also known as palmitoylcarnitine; C16:0). Elevated circulating concentrations of medium‐ to long‐chain acylcarnitines, particularly C16:0, are associated with increased risk of type 2 diabetes mellitus (relative risk = 1.28 [1.05–1.54]; Sun et al., 2020). However, the interpretation of acylcarnitine release depends crucially on the underlying metabolic context. In a catabolic muscle atrophic state following prolonged bed rest, we previously reported a reduction in intramuscular medium‐ to long‐chain acylcarnitine concentrations consistent with impaired mitochondrial fatty acid flux and β‐oxidation capacity (Eggelbusch et al., 2024). In contrast, given our present hypertrophic context, the concomitant uptake of palmitic acid together with release of its carnitine ester suggests active mitochondrial fatty acid flux, with export of β‐oxidation intermediates back into the circulation. This phenomenon has been described during recovery from metabolic stress, when transient acylcarnitine efflux reflects metabolic flexibility rather than mitochondrial insufficiency (Schranner et al., 2020). The lipid‐related metabolite profile 24 h after resistance training might suggest a state of enhanced lipid turnover rather than metabolic dysfunction.

### 4.6 Methodological considerations

We used a reciprocal design aligning in vitro IGF‐1‐stimulated myotube exchanges (fresh vs. spent media) with human in vivo arteriovenous differences. To our knowledge, this is the first study to combine these complementary approaches to map the anabolic metabolome. The cell culture model allows for the isolation of direct signalling effects free from systemic interferences, whereas the human cross‐over design captures the integrated physiological response. We acknowledge that arteriovenous concentration differences reflect the net exchange of the entire vascularized limb, including the skin, adipose tissue and bone, rather than skeletal muscle exclusively. However, by using a unilateral exercise model, in which subjects served as their own controls, we effectively subtracted the systemic background. Consequently, the divergent metabolite exchange observed between the trained and control legs can be attributed to the resistance‐trained musculature. While we acknowledge that arterial abundances are subject to inherent analytical variance, the use of a synchronized sampling protocol, and identical processing of arterial and venous pairs minimizes the impact of systemic fluctuations. Given that the arterial input is shared by both limbs, any significant divergence in venous effluent between the trained and control legs reflects a true limb‐specific metabolic shift rather than systemic or analytical noise.

A limitation of our study was the absence of limb blood flow measurements. Without blood flow data, calculation of absolute uptake and release rates (e.g., in nanomoles per minute) via the Fick principle was not possible, and our results therefore reflect venous‐to‐arterial directionality rather than quantitative metabolic flux. This distinction is biologically important, because two metabolites might show identical arteriovenous concentration differences yet represent vastly different molar fluxes depending on limb perfusion. Given that this methodology was not feasible, our data should therefore be interpreted as a directional, hypothesis‐generating metabolomic screen rather than a quantitative flux analysis. Future studies coupling untargeted arteriovenous metabolomics with blood flow quantification and targeted tracer approaches will be necessary to establish true net metabolite fluxes across the hypertrophying limb.

Furthermore, blood sampling was performed ∼24 h postexercise, in a postabsorptive state. As discussed above, this design choice is likely to have masked potential amino acid exchange that depends on postprandial hyperaminoacidaemia (Tipton et al., 1999), suggesting that, without exogenous substrate availability, the ‘anabolic potential’ of the trained muscle does not translate into substantial net amino acid uptake from the circulation. Furthermore, given the exploratory nature of this study and the inherent interindividual variability in human metabolic responses, our findings might not capture subtle or transient exchanges that require larger cohorts or continuous sampling. Nevertheless, the observed nominal trans‐muscular shifts provide a robust foundation for future investigations into the temporal dynamics of the hypertrophying skeletal muscle, functioning as an ‘anabolic sink’ hypothesis.

Finally, we also acknowledge that C2C12 cells are murine derived, immortalized, and might not fully recapitulate human muscle metabolic responses.

## 5 CONCLUSION

Our data suggest that 24 h after resistance training, the fasted skeletal muscle resides in a state of ‘latent anabolism’, metabolically primed but constrained by insufficient substrate availability. in vivo, we observed a subtle metabolic signature characterized by: (1) sustained anaplerotic demand or non‐essential amino acid synthesis (α‐ketoglutaric acid); (2) elevated lipid turnover, reflected by acylcarnitine release and fatty acid handling; and (3) selective clearance of the polyamine by‐product acisoga. Notably, this nominal profile occurred in the absence of broad amino acid uptake, suggesting that the postexercise anabolic window is permissive but substrate dependent. In contrast, IGF‐1‐stimulated myotubes displayed a robust anabolic exchange programme, marked by coordinated uptake of amino acids and biosynthetic cofactors (including serine, arginine and pyridoxamine), together with enhanced lactate release indicative of glycolytic rerouting. The divergence between these models suggests that resistance‐trained human muscle retains anabolic capacity 24 h postexercise but fails to engage comparable nutrient uptake in postabsorptive conditions despite an activated growth programme.

Future studies should extend this work by coupling high‐resolution arteriovenous metabolomics with controlled postexercise nutrient provision (e.g., timed whey protein pulses) and simultaneous blood flow quantification. Such designs would test directly whether substrate provision unlocks the dormant metabolic fluxes observed in vivo, thereby completing the mechanistic link between nutrient supply, metabolic remodelling and skeletal muscle hypertrophy. Furthermore, targeted validation of putative metabolite annotations, particularly level 2 and level 3 features identified here, using authentic chemical standards and targeted liquid chromatography–tandem mass spectrometry approaches will be essential to substantiate the molecule‐specific mechanistic interpretations suggested by the present untargeted screen.

## AUTHOR CONTRIBUTIONS

Tim Havers: Conceptualization, methodology, investigation, formal analysis, writing—original draft. Moritz Eggelbusch: Conceptualization, methodology, investigation, formal analysis, writing—original draft. Marius Meinhold: Investigation, methodology. Kira Sing: Methodology, investigation. Rainer Okrojek: Investigation. Anna Artati: Investigation, formal analysis. Michael Witting: Investigation, formal analysis. Dominik Lutter: Formal analysis. Claire Kieker: Formal analysis; Writing—review and editing. Collin Starke: Investigation. Tushar More: Investigation. Karsten Hiller: Investigation, formal analysis. Flemming Dela: Writing—review and editing. Martin Schönfelder: Investigation, review and editing. André Kafka: Writing—review and editing. Stephan Geisler: Conceptualization, writing—review and editing. Henning Wackerhage: Conceptualization, writing—review and editing, supervision. All authors have approved the final version of the manuscript and agree to be accountable for all aspects of the work in ensuring that questions related to the accuracy or integrity of any part of the work are appropriately investigated and resolved. All persons designated as authors qualify for authorship, and all those who qualify for authorship are listed.

## CONFLICT OF INTEREST

None declared.

## GENERATIVE AI STATEMENT

During the preparation of this manuscript, we used ChatGPT (OpenAI, free tier; GPT‐5 Instant) to improve the language and readability of text we had written. We used it only for language editing and did not use it to generate scientific content, data, results or their interpretation. We then reviewed and edited the output as needed, and we take full responsibility for the content of this publication.

## Supporting information




**Supplementary Information 1**: Metabolomics data and statistics, in vitro.

**Supplementary Information 2**: Metabolomics data and statistics, in vivo.

## Data Availability

The raw metabolomics data supporting the results presented in this manuscript are provided as Supporting Information (Supplements 1 and 2). All metadata, including experimental conditions and statistical parameters, are included within these files. Any additional data or specific analytical scripts used to generate the figures and tables are available from the corresponding author upon reasonable request.
